# Expression of* TNF-α*,* OPG*,* IL-1β* and the presence of the measles virus RNA in the stapes of the patients with otosclerosis

**DOI:** 10.1007/s00405-014-3008-4

**Published:** 2014-03-28

**Authors:** Małgorzata Potocka-Bakłażec, Monika Sakowicz-Burkiewicz, Jerzy Kuczkowski, Tadeusz Pawełczyk, Czesław Stankiewicz, Wojciech Sierszeń, Zbigniew Jankowski, Jacek Buczny

**Affiliations:** 1 Department of Otolaryngology, Medical University of Gdańsk, Smoluchowskiego17, 80-214 Gdańsk, Poland; 2 Department of Molecular Medicine, Medical University of Gdańsk, Gdańsk, Poland; 3 Department of Forensic Medicine, Medical University of Gdańsk, Gdańsk, Poland; 4 University of Social Sciences and Humanities, Sopot, Poland

**Keywords:** Otosclerosis, Etiopathogenesis, Measles virus, Cytokines

## Abstract

Persistent measles virus infections play a crucial role in the pathomechanism of otosclerosis. The study was undertaken to investigate the role of tumor necrosis factor-α (TNF-α), interleukin 1β (IL-1β) and osteoprotegerin (OPG) in otosclerotic bone remodeling and to assess the relation of *TNF-α, OPG* and *IL-1β* expression levels in otosclerotic stape footplates to the occurrence of measles virus infection. 61 patients with otosclerosis were treated surgically. Thirty-one stapes obtained from cadavers of people, who had died from a sudden cause were used as a control group. The presence of measles virus RNA and the expression levels of *TNF-α, IL-1β* and *OPG* in otosclerotic foci were assessed using one-step RT-PCR. The presence of measles virus RNA was noted in 80.3 % of otosclerotic stapes (49 out of 61) and 9.7 % of normal tissues (3 out of 31). Transcript of *TNF-α, IL-1β* and *OPG* was detected in 40, 46 and 18 virus-positive stapes, respectively. The transcript level of *TNF-α* and *IL-1β* was significantly higher in otosclerotic tissues comparing to normal tissue. The *OPG* expression level was significantly lower in otosclerotic tissues comparing to controls. The presence of measles virus RNA in the stapes may indicate its role in the pathogenesis of otosclerosis. The presence of *TNF-α* and * IL-1β *mRNA in the virus-positive stapes could be the result of viral antigen stimulation and may be a marker of inflammation the otosclerotic focus. The lack of *OPG* mRNA and the presence of *TNF-α* and *IL-1β * mRNA in the majority of otosclerotic tissues reflect the bone remodeling process occurring in the stapes.

## Introduction

Hearing loss in humans significantly decreases quality of life and often leads to social isolation. One of the causes of acquired hearing loss is otosclerosis. The unexplained etiology and pathomechanism of this disorder generates great interest within the international scientific community.

Otosclerosis occurs only in humans and is the cause of 5–9 % of all hearing losses and 18–22 % of those with conductive hearing losses. Approximately 10 % of patients with otosclerosis develop sensorineural hearing loss [1–3]. Women are twice as likely to suffer from otosclerosis than men [1, 4–9]. The onset of the symptoms of this disease occurs mainly in the third decade of life [6, 10]. In the early stages, when the otosclerotic foci are often localized in the anterior or posterior poles of the stape footplate, the conductive hearing loss is the prevailing symptom of the disease [2]. The progress of the otosclerotic process in the other parts of the otic capsule can lead to sensorineural hearing loss, tinnitus and vertigo [7, 11–13].

According to our current knowledge, interacting environmental and genetic factors are the underlining causes of the disease. Measles virus elements have been frequently detected in otosclerotic tissues, so there is a reliable hypothesis about the viral origin of the disorder [11, 14–20]. It is assumed that the main factor involved in the pathogenesis of otosclerosis is the secondary immunological response induced by persistent measles virus infection. The virus can induce the secondary autoimmune reaction via the response of the natural killer (NK) cells, cytotoxic cells activated by lymphokines, CD8+ cells, granulocytes and macrophages [16, 19, 21]. In response to viral or bacterial infections, the cells secrete pro-inflammatory cytokines, e.g. tumor necrosis factor-α (TNF-α) and interleukin-1β (IL-1β). TNF-α is secreted by activated monocytes, macrophages, lymphocytes B and T and osteoclasts [11, 19, 22]. It plays a very important role in bone remodeling and in the maturation of osteoclast precursors. IL-1β is an important mediator of inflammation and is mainly secreted by activated macrophages [22]. It plays a leading role in the bone resorption processes in many metabolic disorders. TNF-α and IL-1β are called osteoclastogenesis factors [23]. The presence of TNF-α in otosclerotic bone indicates osteoclast activity and the existence of inflammation [19]. A rise in TNF-α gene expression results in increasing osteoclast activation and bone resorption [11, 19].

Osteoprotegerin (OPG) is a member of the TNF superfamily and also participates in bone homeostasis. OPG is also known as TNFRSF11B (tumor necrosis factor receptor superfamily member 11B) and its main function is the inhibition of bone resorption. This glycoprotein inhibits maturation and activation of osteoclasts and induces their apoptosis [11, 24–26]. Thus, the activities of TNF-α, IL-1β and OPG lead to contrasting effects concerning the bone remodeling. Action of TNF-α and IL-1β leads to an increase in bone resorption, whereas osteoprotegerin inhibits its destruction [12, 26, 27].

In our study, we analyzed the expression level of proinflammatory cytokines (TNF-α, IL-1β), OPG and the presence of measles virus RNA in fragments of the stapes (superstructure and part of the otosclerotic changed footplate) removed during stapedotomy performed in patients with otosclerosis. Our findings support the assumption on primary role of measles virus infection in pathogenesis of otosclerotic bone remodeling in the stapes.

## Materials and methods

Sixty-one patients suffering from otosclerosis (*N* = 61, male = 21, female = 40), treated surgically in the Department of Otolaryngology, Medical University of Gdańsk, were analyzed prospectively. The otolaryngological preoperative examination consisted of detailed anamnesis and audiological studies. Each patient underwent surgery on the stapes to improve their hearing. During the stapedotomy, the superstructure and part of the stapes footplate were removed and subjected to further molecular analysis. The diagnosis of otosclerosis was based on clinical, audiometric and surgical findings. The assessment of otosclerotic lesions during the surgery was based on the Portmann Classification [28]. The patients had no history of any other ear diseases or head injuries. The control group consisted of 31 stapes taken from persons who had died of a sudden cause. The study was conducted with the consent of the local ethics committee (NKEBN/5/2008), and written consent was obtained from all patients prior to enrollment in the study.

### Extraction of total RNA from bone fragments

After removal, both parts of the stapes from the patients and the stapes from cadavers were transported in RNA stabilization solution (RNA-later, Sigma-Aldrich) to the Department of Molecular Medicine and there the samples were stored at −20 °C until analysis.

The bone fragments were pulverized in a sterile porcelain mortar with 500 μl of TRI Reagent (Sigma-Aldrich). Isolation of total RNA was carried out in accordance with the Chomczyński procedure [29], with our own modifications. TRI reagent and suspended material were vortexed briefly and then left standing for 10 min at 4 °C. Next, chloroform (250 μl) was added, and samples were vigorously shaken, incubated at 4 °C for 15 min and centrifuged (10,000×*g* for 15 min at 4 °C). The upper aqueous phase was removed into a new Eppendorf tube, an equal volume of isopropanol was added and RNA was precipitated via overnight incubation at −20 °C, followed by centrifugation (10,000×*g* for 15 min at 4 °C). RNA pellets were washed first with 96 % and then with 70 % (v/v) ethanol, air-dried and resolved in diethyl-pirocarbonate-treated thermo-sterilized water (11 μl) and stored at −20 °C until further analysis.

### Detection of measles virus RNA

The presence of measles virus RNA in analyzed tissues was detected by qualitative RT-PCR and the use LightCycler RNA Master HybProbe (Roche Diagnostics GmBH Mannheim, Germany). The reaction was performed on a LightCycler 2.0 (Roche Molecular Biochemicals, Mannheim, Germany). The reaction mixture (20 μl) consisted of medium Master HybProbe, 50 ng of total RNA, 3.25 mM Mn[OAc]_2_, 0.5 μM of each primer and 0.2 μM of each probe. The primers and probes used were as described previously [30] and are listed in Table 1. As a negative controls water was run with every PCR. Live, attenuated Schwarz-type measles viruses isolated from Priorix vaccine (GlaxoSmithKline Biologicals SA, Belgium) was used as a positive control in measles virus amplification reactions. The RT-PCR program consisted of 30-min reverse transcription at 61 °C, 30 s denaturation at 95 °C, 40 cycles of 2 s denaturation at 95 °C, 10 s annealing at 60 °C, and 17 s extension at 72 °C. Fluorescence was measured at the end of each annealing step.

**Table 1:** Primer and probe sequences used in RT-PCR

MV	Primers	F	5′-CTTgTTTCAgAgATTgCAATgCAT-3′
		R	5′-ggCCTCTCgCACCTAgTCTAg-3′
	Probes	FL	5′-AAgCCAgggAgAgCTACAgAgAAACC-FL
		LC	640-CCCAgCAgAgCAAgTgATgCgAgA-PH
OPG	Primers	F	5′-AAgggCgCTACCTTgAgATA-3′
		R	5′-CATCTATTCCACATTTTTgAgTTgAT-3′
	Probes	FL	CAATTTgTgTgTTTTCTACAgggTgCTT-FL
		LC	640-AgATgACgTCTCATTTgAgAAgAACCCAT-PH
TNF-α	Primers	F	5′-ACAAgCCTgTAgCCCATgTT-3′
		A	5′-AAgAggACCTgggAgTAgATgA-3′
	Probes	FL	gCATTggCCCggCggTTC-FL
		LC	640-CCACTggAgCTgCCCCTCAgCT-PH
IL-1β	Primers	S	5′-CAgggACAggATATggAgCAA-3′
		A	5′-gCAgACTCAAATTCCAgCTTgTTA-3′
	Probes	FL	gCTTATCATCTTTCAACACgCAggACA-FL
		LC	640-gTACAgATTCTTTTCCTTgAggCCCA-PH
β-Actin	Primers	F	5′-AgCCTCgCCTTTgCCgA-3′
		R	5′-CTggTgCCTggggCg-3′
	Probes	FL	TTgCACATgCCggAgCCgTTg-FL
		LC	705-CgACgACgAgCgCggCgATATC-PH

### Real-time polymerase chain reaction (PCR) analysis

The levels of *TNF*-*α*, *IL*-*1β,* and *OPG* transcripts were analyzed via real-time RT-PCR with the use LightCycler RNA Master HybProbe (Roche Diagnostics GmBH Mannheim, Germany). The reaction was performed in a Light Cycler 2.0 (Roche, Mannheim, Germany). The β-actin transcript was used as an internal standard and was amplified together with each target gene transcript in the same tubes using primers and probes as shown in Table 1.

Thermocycling was conducted at a final volume of 20 μl medium containing: 7.5 μl of Master HybProbe, 3.25 mM Mn[OAc]_2_, 0.5 μM of each PCR primer, 0.2 μM of each hybridization probe and ~50 ng of total RNA isolated from analyzed tissue. The concentration of FL probe for β-actin was 0.4 μM (Table 1). As a negative control, water was run with every PCR assay. The total RNA isolated from the human B lymphocytes cell line SKW 6.4 was used as a positive control for *IL*-*1β* and *TNF*-*α mRNA* amplification reactions. The positive control for the *OPG* mRNA amplification reaction was the total RNA isolated from the osteosarcoma cell line-143 b. The level of each transcript was normalized to that of the β-actin (reference transcript). Analysis of the data was performed using Light Cycler software.

### Statistical analysis

To run quantitative analyses several statistical test were used. Chi-squared statistic for 2 × 2 design was applied. In addition, Cramer’s *V* was used to assess effect size of a relationship between variables in this design. It was assumed that the results between 0.50 and 0.75 indicate moderate effect size. In order to compare two independent groups, a *t* test was performed followed by effect size assessment with Cohen’s *d*. It was assumed that *d* between 0.20 and 0.50 indicate modest effect size, but *d* between 0.50 and 0.80 demonstrate medium effect size.

## Results

The mean age of patients was 43.5 years (range 24–64 years). Female to male ratio was 1.9:1. The air-bone gap at the 1,000 Hz was at least 30 dB, conductive (59 % of patients) or mixed hearing loss (41 % of patients) was associated with the absence of the stapedial reflex on pre-operative audiometry. The average patient’s symptom duration (mainly hearing loss) was 9 years (range 0.5–40). More than half of all cases were classified as type 4 according to the Portmann Classification.

**Fig. 1:**
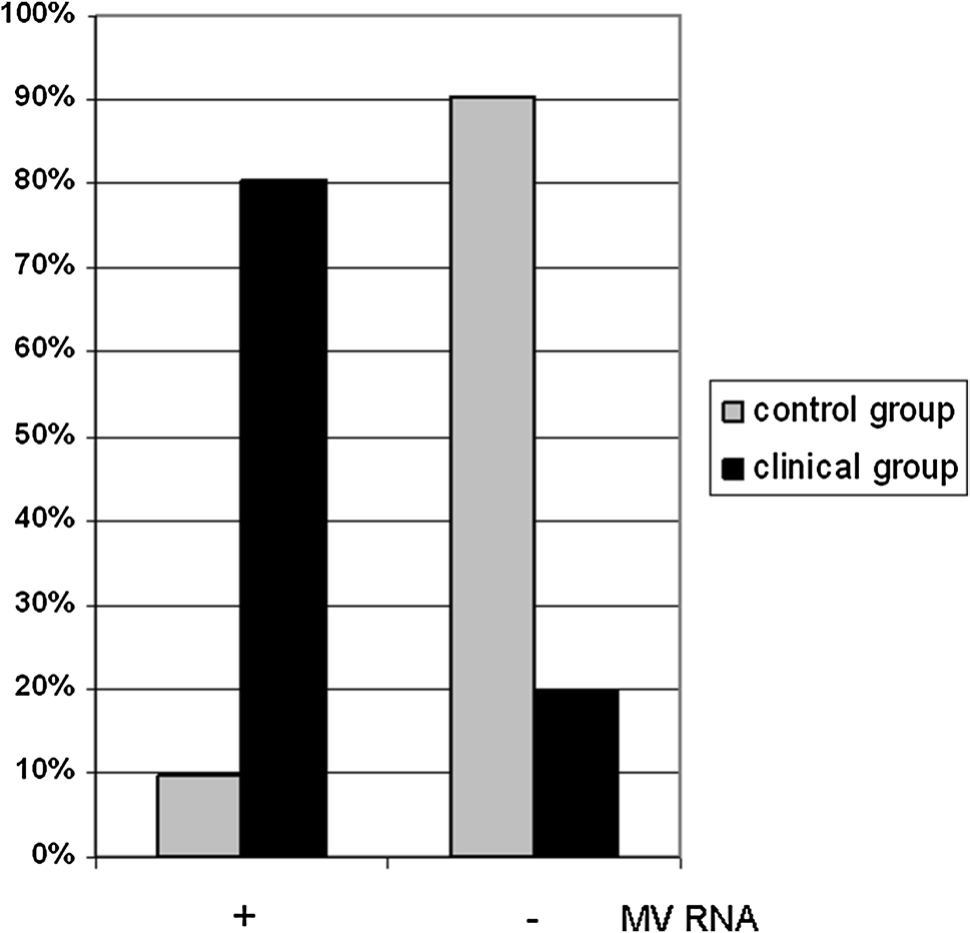
The presence of measles virus RNA in the stapes of patients with otosclerosis and control groups. The incidence of MV RNA was expressed in percentage (%), where 100 % in the control group is n = 31 and in clinical group is n = 61

Measles virus RNA was detected in 49 out of 61 (80.3 %) stapes obtained from patients with otosclerosis. In the stapes from the control group, the presence of measles virus RNA was noted in three cases out of 31 (9.6 %) (Fig. 1). An analysis of the frequency of occurrence of measles virus RNA in patients with otosclerosis and in the control group showed that the incidence of measles virus RNA is much higher in the clinical than in the control group. The analysis demonstrated that the difference in the incidence of measles virus depending on the groups was statistically significant [*χ* ^2^(1.88) = 41.75; *p* < 0.001; *V* = 0.67].

**Fig. 2:**
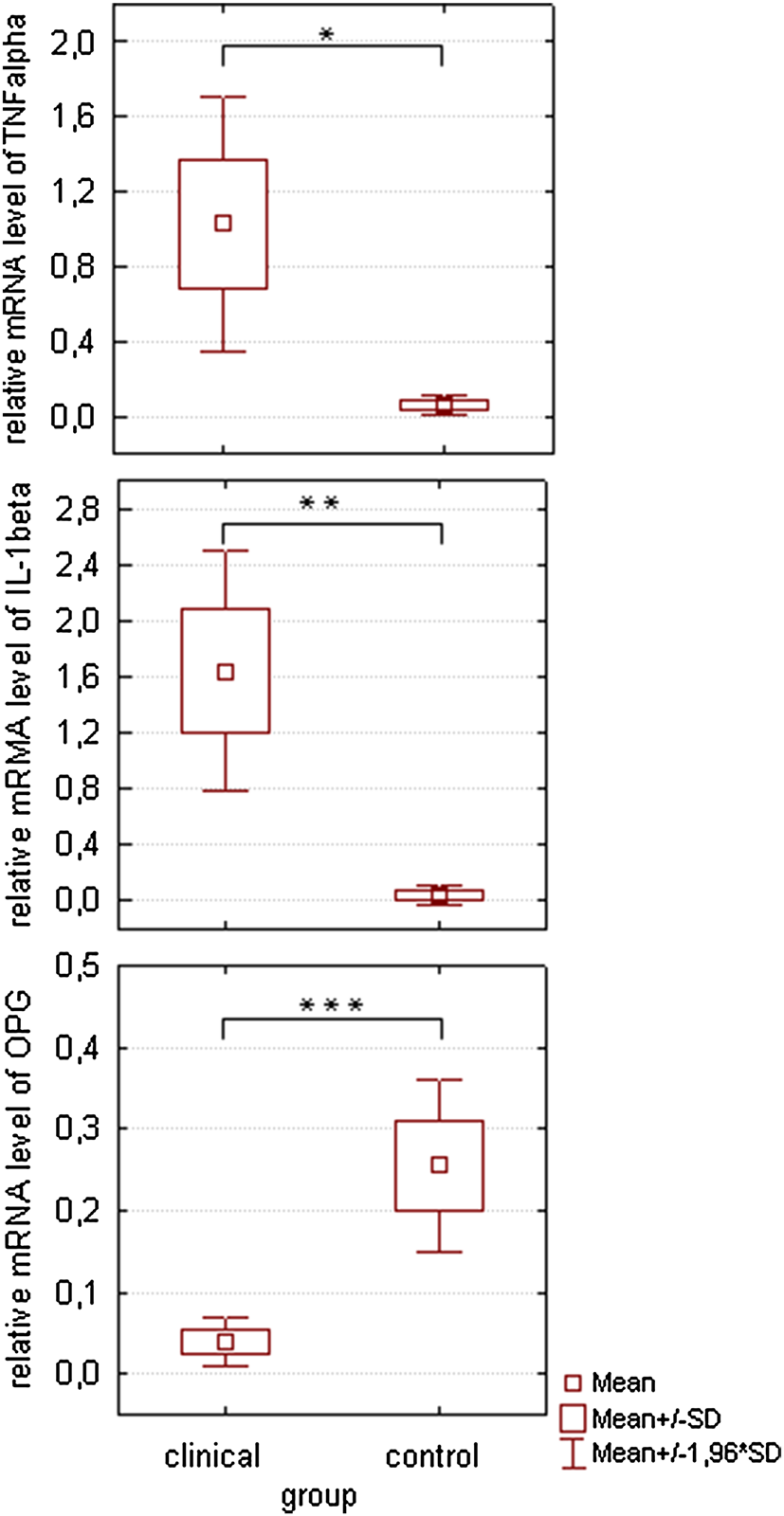
The level of TNF-α, IL-1β and OPG gene transcripts in the stapes of the patients with otosclerosis. To assess the amount of investigated gene transcripts, the multiplex Real-time RT-PCR was performed as described under Material and Methods. The β-actin gene transcript was used as a reference template. Results are expressed as the target/reference ratio of each sample normalized by the target/reference ratio of the suitable calibrator. The data are mean ± SD. *p < 0.05, **p < 0.01, ***p < 0.001

Our analyses showed that *TNF*-*α* transcript was on detectable level in 40 virus-positive (81.6 %) and in 11 virus-negative (91.7 %) bone samples from the clinical group. *IL*-*1β* transcript was detected in 46 virus-positive (93.8 %) and in 10 virus-negative (83.3 %) stapes obtained from patients with otosclerosis *OPG* transcript was detectable in only 18 virus-positive (36.7 %) and in 5 virus-negative (41.7 %) patient’s stapes. In the specimens from cadavers, *OPG* mRNA was detectable in 23 (74.1 %) stapes, *TNF*-*α* mRNA in 8 (25.8 %) stapes and *IL*-*1β* mRNA in 6 (19.3 %) stapes. However, we observed that the TNF-α and IL-1β expression levels were significantly higher in tissue samples obtained from patients with otosclerosis as compared to the control, *t* (<1.96) (Fig. 2).

Conversely, the transcript level of the *OPG* gene was higher in the control group than in the clinical one *t* (=4.74); *p* < 0.001.

The analysis of the value of Cohen’s *d* statistics indicates that the extent of these differences was either small (*d* > 20) or medium (*d* > 50). In the measles virus-positive samples, there was no correlation between the transcript levels for genes *TNF*-*α* and *OPG* and between the transcript levels for genes *IL*-*1β* and *OPG*. It was estimated that the level of *TNF*-α*, IL*-*1β* and *OPG* gene expression in the stapes taken from people suffering from otosclerosis is not dependent on the presence of measles virus RNA (*t* < 1).

## Discussion

We observed the presence of measles virus RNA in 49 stapes taken from patients with otosclerosis. 19.7 % of specimens were free from virus particles. According to Iyer and Gristwood, the histopathological image corresponds to the image of the operating microscope [13]. Owing to the small amount of material available (superstructure and a fragment of the stapes plate) and taking into consideration Iyer and Gristwood’s publication, we did not perform a histological assessment of the stapes. Stapedectomy is not performed in our department due to the significant risk of complications, which can occur after removal of the whole stapes footplate. The main aim of a stapedotomy (a partial removal of the stapes) is an improvement in patient hearing without exposure to additional complications. The stapes fragments, superstructure and part of the otosclerotical changed footplate that were removed during a stapedotomy are relatively small. After histological assessment, these samples would not be sufficient to examine the presence of measles virus RNA and to analyze the level of *TNF*-*α, IL*-*1β* and *OPG* expression.

The results of our research can be compared with the reports of Karosi et al. [18–20, 31, 32]. They detected measles virus RNA with 100 % effectiveness when they conducted histological examinations [20, 31] or with 60 % effectiveness without histological confirmation of the otosclerosis [18, 19, 32]. In our study, we detected measles virus RNA in 80.3 % of the stapes. Therefore, we can conclude that a definitive diagnosis of otosclerosis cannot be based only on preoperative examinations, such as anamnesis, otoscopy, audiometric findings or on the image under the operating microscope. Furthermore, in case of some patients without viral RNA in the stapes, otosclerosis can have a genetic origin. Moreover, real-time PCR is a much more precise and sensitive method than RT-PCR (reverse transcriptase polymerase chain reaction) (80.3 % vs. 59–62.3 %) in the assessment of the presence of measles virus RNA in stapes. We analyzed only fragments of the stapes collected during the stapedotomy, so it was not possible to verify how extensive was the part of the otosclerotic focus removed with the footplate fragment. In some cases, the part of the removed plate was very small. It is difficult to verify if this fragment had otosclerotic foci. Nevertheless, we obtained similar results to Karosi et al. in terms of the assessment of the presence of measles virus RNA in the stapes. Our study confirms the superiority of real-time PCR that is significantly more sensitive (10×) than RT-PCR. Moreover, taking into account the fact that most of the material removed during surgery was the superstructure of the stapes, we cannot ignore the possibility that measles virus RNA may be present in the other parts of stapes than footplate. There is an assumption that virus particles may also be present beyond the footplate and in the crus of the stapes or in other structures of the middle ear which can be detected only using methods as sensitive as real-time PCR.

It should be stressed that although several reports employing various methodologies documented some relations of measles virus infection with development of otosclerosis, there are also studies showing lack of viral RNA in samples from patients with otosclerosis. Grayeli et al. [33] did not find measles virus RNA in the examined samples of otosclerotic tissue. However, they also did not detect GAPDH mRNA in 8 of 30 stapes, so RNA was present only in 73 % of the examined samples and such a low efficacy of RNA extraction would indicate relatively high degree of RNA degradation. Authors of that study do not provide information on the transportation and storage conditions of the otosclerotic tissue samples. Appropriate sample handling conditions are crucial for obtaining reliable results. Moreover, stability of measles virus RNA may be lower than other RNA particles in the samples. Another difficulty while dealing with organic samples is sensitivity of the PCR method, which is lower when compared with real-time PCR. Recently another study by Komune et al. [34] reported no sign of measles virus infection in stapes from Japanese patients with otosclerosis. Authors of that study mentioned that autoimmunity and genetic factors could play a role in triggering otosclerotic lesion. They suppose that it is also possible that measles virus infection may initiate the pathological process that can cause otosclerosis even after elimination of measles virus. This assumption is in line with our observations indicating that fibroblasts isolated from otosclerotic lesions are abnormally sensitive to stimulation and release high level of cytokines (unpublished results).

The pro-inflammatory cytokines TNF-α, and IL-1β cause increased inflow of immature forms of osteoclasts from bone marrow into the blood and then into the middle ear, where the inflammation occurs. In contrast to OPG, TNF-α and IL-1β have an anti-apoptotic effect on osteoclasts and thus prolong osteoclast life and increase bone resorption [35]. Karosi et al. [19] detected the presence of TNF-α mRNA using the RT-PCR method and investigated its connection with the presence of measles virus RNA in the otosclerotic foci. They detected the expression of *TNF*-*α* in 88 out of 99 measles virus RNA positive otosclerotic samples (88.8 %) and in 8 without viral RNA.

We achieved results similar to Karosi et al. [11, 19] (81.6 vs. 88.8 %). In addition, we investigated the expression level of *IL*-*1β.* The presence of the *IL*-*1β* transcript was recorded in 93.8 % of the virus-positive otosclerotic samples. We used the real-time PCR method, which, apart from the qualitative analysis of the genetic material, allows the determination of the quantity of the analyzed material initially present in the tested sample. Thus, we checked if there is a connection between the levels of *TNF*-*α, IL*-*1β, OPG* expression and the presence of measles virus RNA in the otosclerotic stapes. It transpired that the level of expression for these genes in the stapes removed from people suffering from otosclerosis does not depend on the presence of the viral RNA. However, more essential seems to be the presence or absence of their mRNAs in the otosclerotic focus.

We assume that the lack of *OPG* and the presence of the *TNF*-*α* and *IL*-*1β* in the otosclerotic stapes simultaneously indicates the suppression of *OPG* due to increased levels of the pro-inflammatory cytokines. The presence of *OPG* mRNA and the lack of *IL*-*1β* and *TNF*-*α* mRNA in most of the stapes removed from healthy people who had died a sudden death might confirm our assumption.
